# Advances in Systemic Sclerosis: From Pathogenetic Pathways toward Novel Therapeutic Targets

**DOI:** 10.3390/life13020513

**Published:** 2023-02-13

**Authors:** Eloisa Romano, Irene Rosa, Mirko Manetti

**Affiliations:** 1 Section of Internal Medicine, Department of Experimental and Clinical Medicine, University of Florence, 50134 Florence, Italy; 2 Section of Anatomy and Histology, Department of Experimental and Clinical Medicine, University of Florence, 50134 Florence, Italy

Systemic sclerosis (SSc, or scleroderma) is a multifaceted rare connective tissue disease. SSc pathogenesis is defined by early microvascular impairment, perivascular inflammation, and immune dysregulation, which evolve into both cutaneous and visceral organ fibrosis, a process orchestrated in the main by myofibroblasts [1,2]. Microvascular damage may precede fibrosis by months or even years and clinically manifests with Raynaud’s phenomenon (RP), telangiectasias, pitting scars, characteristic periungual microvascular abnormalities detectable by nailfold videocapillaroscopy (NVC), ischemic digital ulcers (DUs), pulmonary arterial hypertension (PAH), and scleroderma renal crisis [1]. SSc has a chronic progression and significantly affects the length and quality of a patient’s life, with internal organ complications, in particular interstitial lung disease (ILD) and primary heart involvement, being mostly responsible for the high mortality rate. In addition, the disease represents a real social burden owing to its heavy impact on facial appearance, orofacial functions, and everyday life activities [3]. Although the etiology of SSc remains unknown, a large body of evidence now indicates that genetic susceptibility factors have an essential role in the onset and the evolution of this disease [4]. In recent years, there have been substantial advances in the understanding of SSc pathogenesis, as well as in early diagnosis and clinical management of patients. In this context, in vitro studies, together with the establishment of several animal models for SSc, have represented powerful research tools in the mission to decipher the cellular and molecular mechanisms underlying endothelial cell damage, uncontrolled fibroblast activation/differentiation into profibrotic myofibroblasts, and dysfunctional immune response with the production of different kinds of autoantibodies [5,6]. Since SSc manifestations are extremely heterogeneous among patients, the discovery and validation of novel reliable biomarkers may help to identify disease risk, improve early diagnosis and prognosis, and assess patient responses to therapy. In such a scenario, the collected contributions of this Special Issue of *Life*, entitled “Advances in Systemic Sclerosis: From Pathogenetic Pathways toward Novel Therapeutic Targets” and consisting of nine original articles and three reviews, are extremely pertinent, covering different aspects of SSc pathophysiology and clinical manifestations.

In this collection, Kosałka-Węgiel and colleagues present very interesting results of a genetic association study investigating four single nucleotide polymorphisms (i.e., rs361525, rs1800629, rs1799964, and rs1799724) located within the promoter of the *TNFA* gene in a SSc cohort compared to a control group [7]. In this original contribution, the authors found an association between the C rs1799724 allelic variant and an increased risk of SSc. In addition, they reported that the CT rs1799964 and AG rs361525 genotypes might enhance cancer susceptibility in SSc patients [7]. Furthermore, it was found that patients with the AG rs1800629 genotype had a higher prevalence of anti-Ro52, a type of antibodies that have been associated with a higher risk of developing different kinds of cancers, particularly those affecting the digestive and female reproductive systems [7,8]. Of note, since this study consists of a preliminary case-control research performed on a relatively small sample group, the validation of the results in larger SSc cohorts will be required.

In another original work drawing from the bases of previously reported associations between SSc and oropharyngeal manifestations, including microstomia, limited mouth opening, clinical attachment loss (CAL) as the primary manifestation of periodontal disease, and xerostomia [9], Buchbender et al. correlated the expression of inflammatory mediators in SSc biofilm samples with different clinical parameters of periodontitis [10]. In such an innovative study, SSc patients showed significantly increased periodontal parameters, such as CAL and bleeding on probing, gingival recession, cervical defects, and decreased mouth opening. Moreover, the authors reported higher concentrations of interleukin (IL)-6, matrix metalloproteinase (MMP)-9, and CD90 in the biofilm samples, as well as a significant positive correlation of IL-6/MMP-9 and a negative correlation between mouth opening/CAL and IL-6/CAL. These results led the researchers to conclude that the peculiar inflammatory microenvironment could have an impact on the prevalence of periodontitis in SSc patients [10].

The topic of SSc orofacial manifestations has also been addressed in a retrospective study conducted by Berl et al., in which the authors described their experience and results with total facial autologous fat grafting in SSc patients. To perform this, the authors had made use of a technique consisting of the subcutaneous injection of fat in all regions of the face, including the superficial temporal fascia and superficial muscular aponeurotic system [11]. In the treated SSc patients, the oral opening increased by an average of 33%, and all of them reported improvement in quality of life and satisfaction with the aesthetic outcomes [11]. Interestingly, patients who underwent multiple surgeries were able to be injected with increasing amounts of fat over time, indicating that the treatment may led to improved facial tissue elasticity [11].

Given that iron overload predisposes the targeted tissues to the formation of reactive oxygen species (ROS), and that both iron and ROS have been implicated in profibrotic myofibroblast generation and persistence, Sfikakis and colleagues hypothesized that erythrocyte-derived iron uptake by tissue resident cells could be involved in the development of fibrosis [12]. In their original paper, these scholars reported the occurrence of iron-induced oxidative stress and DNA damage in fibroblasts in vitro, as well as in peripheral blood mononuclear cells ex vivo [12]. Moreover, an increased iron deposition in the fingers of a patient with early SSc and nailfold microhemorrhages could be detected by using magnetic resonance imaging [12]. Based on these preliminary findings, the authors proposed that microvasculopathy-related iron uptake by resident endothelial cells, fibroblasts, and infiltrating leukocytes may be an additional trigger of oxidative stress and damage in SSc, thus representing a novel pathogenetic link between microvasculopathy and fibrosis, as well as a potential treatment target that deserves further investigation [12].

Although liver involvement in SSc is considered atypical, the occurrence of portal hypertension and, more specifically, of the syndromes called idiopathic portal hypertension (IPH) and regenerative nodular hyperplasia, has been anecdotally reported in the literature for SSc patients. In this context, Colaci et al. contributed an interesting case report to this Special Issue of a female patient with the limited cutaneous SSc (lcSSc) subset, anti-centromere antibodies and no previous significant visceral involvement. This patient, after being admitted to the hospital because of the onset of ascites, was diagnosed with IPH [13]. After the detailed description of their case, the authors also performed a revision of the literature, identifying other similar cases and underscoring that, even though it is a rare condition, the onset of IPH in SSc patients should not be overlooked [13].

Based on the evidence that SSc patients often present cardiovascular autonomic dysfunction, a state associated with the risk of arrhythmic complications and mortality, and that little is known regarding its progression over time, Rodrigues and coworkers conducted a five-year follow-up study in order to investigate the progression of cardiac autonomic impairment in lcSSc and diffuse cutaneous SSc (dcSSc) patients in comparison to a healthy control group [14]. When compared to controls, all patients showed a sympatho-vagal imbalance consisting of sympathetic predominance and vagal withdrawal both at baseline and over time [14]. Moreover, the sympatho-vagal dysfunction worsened only in patients with the more severe phenotype of the disease, namely the dcSSc subset [14]. On the basis of their findings, the authors suggested that cardiac autonomic control could represent a potential therapeutic target in SSc, and that pharmacological and non-pharmacological approaches capable of stimulating the vagus nerve and reducing sympathetic activity should be taken into consideration to counteract the autonomic dysfunction in these patients [14].

Substantial contributions to this Special Issue were additionally made by three original papers which aimed to identify novel serum biomarkers capable of assessing the evolution of the SSc pathological process and predicting the disease prognosis and the response to therapy [15,16,17].

In the first submission from this group of original articles, Kawanabe et al. investigated circulating levels of C-X-C chemokine ligand 1 (CXCL1), reporting not only significantly increased values of such chemokine in SSc patients, but also an association with the activity of SSc-related ILD [15]. Furthermore, higher serum CXCL1 levels were shown to be predictors of SSc-related ILD responses to the anti-CD20 monoclonal antibody drug rituximab, which targets B cells [15].

The second contribution is represented by the article of Romano et al., in which the authors demonstrated an increase in the levels of serum soluble junctional adhesion molecule (sJAM)-A and sJAM-C in SSc patients, especially in those featuring early/active NVC patterns and ischemic DUs [16]. In addition, sJAM-C showed a good diagnostic accuracy in discriminating patients from healthy controls and was revealed to be a better biomarker than sJAM-A for SSc-related DUs [16].

In the third study, the same authors measured the serum levels of three neurovascular guidance molecules, namely soluble neuropilin 1 (sNRP1), semaphorin 3E (Sema3E), and Slit2, in a large cohort of SSc patients, focusing on their possible correlation with peripheral vascular disease clinical features [17]. In particular, they demonstrated that: (i) sNRP1 is significantly decreased, with lower sNRP1 serum levels being correlated with the severity of NVC abnormalities and the presence of ischemic DUs; (ii) both Sema3E and Slit2 are increased, with Sema3E better reflecting early NVC abnormalities; and (iii) higher Sema3E correlates with the absence of DUs, while augmented Slit2 associates with the presence of DUs [17]. In addition, both serum sNRP1 and Sema3E showed a moderate diagnostic accuracy, and logistic regression analysis allowed the researchers to identify them as more suitable independent biomarkers to reflect the activity and severity of SSc-related peripheral microvasculopathy [17].

Mandujano and Golubov contributed to this collection with a narrative review in which they summarized the most widely used animal models for SSc [18]. However, since there is to date no experimental model able to recreate RP, a key feature in SSc, they also evaluated, for the first time, the possibility of studying microcirculation in the nailfold of guinea pigs. This was performed with the aim of developing a novel animal model for SSc-related peripheral microvasculopathy including RP and characteristic NVC abnormalities [18].

In another review, Kanno and Shu focused on the main biological functions of the serine protease inhibitor α2-antiplasmin, a molecule that rapidly inactivates plasmin on the fibrin clots or in the circulation [19]. Since α2-antiplasmin has been associated with immune system regulation, angiogenesis, vascular remodeling, and fibrosis, the authors comprehensively discussed how changes in its expression and activity may contribute to SSc progression, and that its blockade may represent a novel therapeutic approach in the treatment of SSc patients [19].

Finally, Fiorentini et al. undertook a narrative review on the potential role of Janus kinase (JAK) inhibitors in the management of SSc-associated ILD [20]. The authors overviewed the current rationale, supporting that, owing to their ability to inhibit the fibrotic process and their anti-inflammatory properties, JAK inhibitors could represent an interesting therapeutic option in the treatment of SSc-associated ILD [20]. Of note was the fact that JAK inhibitors were also shown to exert a potential effect on PAH [20].

In aggregate, the contributions published within the Special Issue of *Life*, entitled “Advances in Systemic Sclerosis: From Pathogenetic Pathways toward Novel Therapeutic Targets”, are excellent examples of the recent advances made in the fields of clinical and translational SSc research. Making continuous progress in the development of our knowledge of the cellular and molecular signatures underlying the development and progression of SSc will be crucial in gaining insights into their potential clinical relevance as biomarkers and/or therapeutic targets and, hence, in making fundamental strides toward precision medicine for this currently incurable disease. We thank all the authors and reviewers for their valuable work and the time they dedicated to providing these excellent contributions to this collection. The second edition of this Special Issue is now accepting the submission of original manuscripts and reviews which address any aspects of SSc research.

## Data Availability

Not applicable.

## References

[1] Asano Y. (2020). The pathogenesis of systemic sclerosis: An understanding based on a common pathologic cascade across multiple organs and additional organ-specific pathologies. J. Clin. Med..

[2] Romano E., Rosa I., Fioretto B.S., Matucci-Cerinic M., Manetti M. (2022). The role of pro-fibrotic myofibroblasts in systemic sclerosis: From origin to therapeutic targeting. Curr. Mol. Med..

[3] Rosa I., Romano E., Fioretto B.S., Matucci-Cerinic M., Manetti M. (2021). Adipose-derived stem cells: Pathophysiologic implications vs therapeutic potential in systemic sclerosis. World J. Stem Cells.

[4] Villanueva-Martín G., Martín J., Bossini-Castillo L. (2022). Recent advances in elucidating the genetic basis of systemic sclerosis. Curr. Opin. Rheumatol..

[5] Varga J., Trojanowska M., Kuwana M. (2017). Pathogenesis of systemic sclerosis: Recent insights of molecular and cellular mechanisms and therapeutic opportunities. J. Scleroderma Relat. Disord..

[6] Yue X., Yu X., Petersen F., Riemekasten G. (2018). Recent advances in mouse models for systemic sclerosis. Autoimmun. Rev..

[7] Kosałka-Węgiel J., Lichołai S., Dziedzina S., Milewski M., Kuszmiersz P., Rams A., Gąsior J., Matyja-Bednarczyk A., Kwiatkowska H., Korkosz M. (2022). Genetic association between TNFA polymorphisms (Rs1799964 and Rs361525) and susceptibility to cancer in systemic sclerosis. Life.

[8] Zhu Y., Tang B., Kang M., Xiao Q. (2022). Anti-Ro52-positive antisynthetase syndrome (ASS): A case report and review of the literature. Ann. Transl. Med..

[9] Smirani R., Truchetet M.E., Poursac N., Naveau A., Schaeverbeke T., Devillard R. (2018). Impact of systemic sclerosis oral manifestations on patients’ health-related quality of life: A systematic review. J. Oral Pathol. Med..

[10] Buchbender M., Lugenbühl A., Fehlhofer J., Kirschneck C., Ries J., Lutz R., Sticherling M., Kesting M.R. (2021). Investigation of the expression of inflammatory markers in oral biofilm samples in patients with systemic scleroderma and the association with clinical periodontal parameters-A preliminary study. Life.

[11] Berl A., Shir-Az O., Perk N., Levy A., Levy Y., Shalom A. (2022). Total facial autologous fat grafting for treating skin manifestations in scleroderma. Life.

[12] Sfikakis P.P., Vlachogiannis N.I., Ntouros P.A., Mavrogeni S., Maris T.G., Karantanas A.H., Souliotis V.L. (2022). Microvasculopathy-related hemorrhagic tissue deposition of iron may contribute to fibrosis in systemic sclerosis: Hypothesis-generating insights from the literature and preliminary findings. Life.

[13] Colaci M., Aprile M.L., Sambataro D., Sambataro G., Malatino L. (2022). Systemic sclerosis and idiopathic portal hypertension: Report of a case and review of the literature. Life.

[14] Rodrigues G.D., Carandina A., Scatà C., Bellocchi C., Beretta L., da Silva Soares P.P., Tobaldini E., Montano N. (2022). Sympatho-vagal dysfunction in systemic sclerosis: A follow-up study. Life.

[15] Kawanabe R., Yoshizaki A., Matsuda K.M., Kotani H., Hisamoto T., Norimatsu Y., Kuzumi A., Fukasawa T., Ebata S., Yoshizaki-Ogawa A. (2022). Serum C-X-C Chemokine Ligand 1 levels in patients with systemic sclerosis: Relationship of clinical and laboratory observations to anti-CD20 monoclonal antibody administration. Life.

[16] Romano E., Rosa I., Fioretto B.S., Matucci-Cerinic M., Manetti M. (2022). Increased circulating soluble junctional adhesion molecules in systemic sclerosis: Association with peripheral microvascular impairment. Life.

[17] Romano E., Rosa I., Fioretto B.S., Matucci-Cerinic M., Manetti M. (2022). Circulating neurovascular guidance molecules and their relationship with peripheral microvascular impairment in systemic sclerosis. Life.

[18] Mandujano A., Golubov M. (2022). Animal models of systemic sclerosis: Using nailfold capillaroscopy as a potential tool to evaluate microcirculation and microangiopathy: A narrative review. Life.

[19] Kanno Y., Shu E. (2022). A2-antiplasmin as a potential therapeutic target for systemic sclerosis. Life.

[20] Fiorentini E., Bonomi F., Peretti S., Orlandi M., Lepri G., Matucci Cerinic M., Bellando Randone S., Guiducci S. (2022). Potential role of JAK inhibitors in the treatment of systemic sclerosis-associated interstitial lung disease: A narrative review from pathogenesis to real-life data. Life.

